# Impact of smoking history on the association between Eicosapentaenoic acid to arachidonic acid ratio and acute coronary syndrome: A multicenter cross-sectional study

**DOI:** 10.18332/tid/84973

**Published:** 2018-03-16

**Authors:** Yuji Nishizaki, Kazunori Shimada, Shigemasa Tani, Takayuki Ogawa, Jiro Ando, Masao Takahashi, Masato Yamamoto, Tomohiro Shinozaki, Tetsuro Miyazaki, Katsumi Miyauchi, Ken Nagao, Atsushi Hirayama, Michihiro Yoshimura, Issei Komuro, Ryozo Nagai, Hiroyuki Daida

**Affiliations:** 1 Department of Cardiovascular Medicine, Juntendo University Graduate School of Medicine, Tokyo, Japan; 2 Department of Cardiology, Nihon University Hospital, Tokyo, Japan; 3 Division of Cardiology, Department of Internal Medicine, The Jikei University School of Medicine, Tokyo, Japan; 4 Department of Cardiovascular Medicine, Graduate School of Medicine, The University of Tokyo, Tokyo, Japan; 5 Department of Internal Medicine, Tokyo Takanawa Hospital, Tokyo, Japan; 6 Department of Biostatistics, School of Public Health, The University of Tokyo, Tokyo, Japan; 7 Division of Cardiology, Department of Medicine, Nihon University School of Medicine, Tokyo, Japan; 8 Jichi Medical University, Shimotsuke-shi, Tochigi-ken, Japan

**Keywords:** acute coronary syndrome, arachidonic acid, eicosapentaenoic acid, EPA/AA ratio, smoking history

## Abstract

**INTRODUCTION:**

The association among smoking history, eicosapentaenoic acid (EPA) to arachidonic acid (AA) ratio and acute coronary syndrome (ACS) is yet to be investigated. The present study aimed to clarify the association between the EPA/AA ratio and ACS prevalence in patients admitted to the cardiology department based on their smoking history.

**METHODS:**

We enrolled 1733 patients from five cardiology divisions located in Tokyo, Japan, and measured their levels of polyunsaturated fatty acids, including EPA and AA, from January 2004 to May 2011. We assessed the association between the EPA/AA ratio and ACS in the subgroups stratified according to smoking history (never, former, current smokers) using multivariate logistic models.

**RESULTS:**

A high EPA/AA ratio was significantly associated with decreased odds of ACS among patients without a smoking history (adjusted odds ratio AOR=0.20, 95% CI: 0.04–0.86) but not in patients with a smoking history (former smoker, AOR=1.50, 95% CI: 0.44–5.03; current smoker, AOR=3.73, 95% CI: 0.34–40.6).

**CONCLUSIONS:**

The EPA/AA ratio and ACS occurrence were found to be significantly associated in patients without a smoking history; however, no such association existed in patients with a smoking history.

**ABBREVIATIONS:**

AA: arachidonic acid, ACS: acute coronary syndrome, CVD: cardiovascular disease, DGLA: dihomo-gamma-linolenic acid, DHA: docosahexaenoic acid, EPA: eicosapentaenoic acid, JELIS: Japan EPA Lipid Intervention Study, PUFA: polyunsaturated fatty acid, RAS: renin angiotensin system, TG: triglyceride.

## INTRODUCTION

Smoking is associated with the development of cardiovascular disease (CVD) and adversely affects the clinical prognosis^1^. Although the mechanisms underlying the association between smoking and the development of CVD are still unclear, smoking-induced vascular inflammation and subclinical vascular damage are believed to play important roles in the development of subsequent clinical CVD events^2-3^.

The eicosapentaenoic acid (EPA) to arachidonic acid (AA) ratio is a known risk factor for acute coronary syndrome (ACS)^4-5^. EPA provides protection against CVD by reducing the triglyceride (TG) levels, inhibiting platelet aggregation, improving vascular endothelial function, and producing anti-inflammatory and anti-hypertensive effects^6^. However, the association among the smoking history, EPA/AA ratio, and ACS risk, warrants investigation. The present study aimed to clarify the association between the EPA/AA ratio and ACS prevalence in patients admitted to the cardiology department based on their smoking history.

## METHODS

This was a multicenter cross-sectional study that was conducted at five cardiology departments located in Tokyo, Japan. The characteristics and lifestyles of the visiting patients were representative of urban Tokyo, because the five participating hospitals included university hospitals and large-size private hospitals in this city. A detailed description of the study population and measured parameters has been previously reported^5,7^. We enrolled 1733 patients and determined their serum polyunsaturated fatty acid (PUFA) levels from January 2004 to May 2011. The enrolled patients lived in urban areas. Acute myocardial infarction was defined as an increased creatine kinase MB fraction or troponin T in patients with ischemic symptoms and/or typical electrocardiographic ST elevation. Unstable angina was defined as angina at rest or accelerated exertional angina combined with typical electrocardiographic ST-changes and an increased requirement for anti-ischemic therapy^5,7,8^. Exclusion criteria were: hemodialysis, EPA intake, the presence of congestive heart failure, and severe liver dysfunction; or other systemic diseases, including malignancy and connective tissue disease. We assessed their clinical background, including coronary risk factors, and evaluated their serum EPA, docosahexaenoic acid (DHA), dihomo-gammalinolenic acid (DGLA), and AA levels. Serum PUFA levels were measured at an external laboratory (SRL, Inc., Tokyo, Japan). Blood samples were collected from patients either during admission or at the laboratory of the outpatient clinic. The present study was approved by the Institutional Ethics Committees of each hospital, and all participants provided informed consent.

We assessed the association between the EPA/AA ratio and prevalence of ACS in the patient subgroups, stratified according to their smoking history (never, former, current smokers) using logistic models adjusted for the following variables: age, gender, body mass index, hypertension, diabetes mellitus, dyslipidemia, family history, estimated glomerular filtration rate; and concomitant use of medications, including antiplatelet agents, statin, calcium channel blockers, beta blockers, renin angiotensin system (RAS) inhibitors (angiotensin converting enzyme inhibitors or angiotensin II receptor blockers), and hypoglycemic agents. The prevalence adjusted odds ratios (AOR) for ACS, between the EPA/AA ratios of the smoking subgroups, were compared using the approximate formula for testing interaction^9^. The alpha level for the statistical tests was set at 5%, and all confidence intervals were at 95% level. All analyses were performed using SPSS Statistics for Windows, Version 22.0 (Armonk, NY) in 2016.

## RESULTS

Table 1 shows the baseline clinical characteristics of the subjects according to their smoking history. Significant differences were observed in the following parameters: 74.8% of those who never smoked, 74.4% of those who formerly smoked and 66.8% of those who were current smokers (p=0.01) were hypertensive; 35.6% of never smokers, 43.5% of former smokers and 43.8% current smokers had diabetes (p<0.01); EPA (μg/dL) levels were 76.0 ± 47.2 for never smokers, 72.9 ± 37.7 for former smokers and 69.2 ± 42.0 for current smokers (p=0.04). There was no significant difference in the EPA/AA ratio of never smokers (0.50 ± 0.32), former smokers (0.50 ± 0.27) and current smoker (0.46 ± 0.30) (p=0.07). From among 1733 consecutive patients, we obtained data from 151 patients with ACS (151/1733, 8.7%) and 1582 patients without ACS (1582/1733, 91.3%). The ACS prevalence of the three groups was significantly different. Table 2 shows the association among the EPA/AA ratio, EPA, and AA and ACS, for each smoking history subgroup. It is noteworthy that a high EPA/AA ratio was significantly associated with decreased odds of ACS among patients without a smoking history (AOR=0.20, 95% CI: 0.04–0.86) but not in patients with a smoking history (former smoker, AOR=1.50, 95% CI: 0.44–5.03; current smoker, AOR=3.73, 95% CI: 0.34–40.6). Interaction tests indicated that the adjusted odds ratio in nonsmokers significantly differed from that in former smokers (AOR=7.5; p=0.03) and current smokers (AOR=18.7; p=0.04), while the adjusted odds ratios did not significantly differ between the former and current smokers (AOR=2.5; p=0.50).

**Table 1:** Baseline clinical characteristics according to smoking history

	*Never smoker*	*Former smoker*	*Current smoker*	
	*(n = 952 )*	*(n = 441 )*	*(n = 340 )*	*p*
Age (years)				
Male	65.8%	94.8%	89.4%	<0.01*
BMI (kg/m2)	24.3 ± 3.8	24.4 ± 2.9	24.7 ± 3.3	0.12
Hypertension	74.8%	74.4%	66.8%	0.01*
Diabetes mellitus	35.6%	43.5%	43.8%	<0.01*
Dyslipidemia	68.2%	74.8%	70.9%	0.04*
Family history of IHD	13.3%	28.8%	19.7%	<0.01*
Total cholesterol (mg/dL)	191.7 ± 36.1	184.3 ± 33.4	194.9 ± 37.0	<0.01*
Triglycerides (mg/dL)	141.1 ± 90.3	141.2 ± 75.1	172.2 ± 125.8	<0.01*
LDL cholesterol (mg/dL)	110.0 ± 30.1	111.5 ± 31.1	114.6 ± 32.7	0.13
HDL cholesterol (mg/dL)	55.1 ± 16.9	45.5 ± 13.3	48.0 ± 15.7	<0.01*
Hemoglobin A1c (%)	6.1 ± 0.9	6.5 ± 1.2	6.4 ± 1.4	<0.01*
eGFR (mL/min/1.73 m2)	68.4 ± 17.8	66.1 ± 16.6	72.4 ± 17.8	<0.01*
EPA (μg/dL)	76.0 ± 47.2	72.9 ± 37.7	69.2 ± 42.0	0.04*
DHA (μg/dL)	142.4 ± 52.7	146.2 ± 51.0	142.8 ± 55.1	0.43
DGLA (μg/dL)	32.9 ± 12.0	31.8 ± 11.2	36.1 ± 13.9	<0.01*
AA (μg/dL)	159.0 ± 42.9	152.5 ± 64.8	159.3 ± 43.1	0.05
EPA/AA	0.50 ± 0.32	0.50 ± 0.27	0.46 ± 0.30	0.07
DHA/AA	0.94 ± 0.41	1.00 ± 0.37	0.93 ± 0.35	0.01*
Statins	51.7%	60.3%	47.6%	<0.01*
Antiplatelet agents	51.3%	87.1%	63.2%	<0.01*
RAS inhibitors	51.7%	52.2%	46.2%	0.17
Calcium channel blockers	50.2%	42.9%	36.5%	<0.01*
Beta blockers	33.8%	51.2%	35.6%	<0.01*
Hypoglycemic agents	18.8%	24.3%	23.2%	0.03*
ACS events	6.2%	12.5%	10.9%	<0.01*

* indicates statistical significance.

Notes: Values are presented as mean ± standard deviation or percentages. The p values were calculated using the chi-squared test (for binary variables) or analysis of variance (for continuous variables). BMI: body mass index, IHD: ischemic heart disease, LDL: low-density lipoprotein, HDL: high-density lipoprotein, eGFR: estimated glomerular filtration rate, RAS: renin angiotensin system, EPA/AA: eicosapentaenoic acid to arachidonic acid ratio, DHA/AA: docosahexaenoic acid to arachidonic acid ratio, DGLA: dihomo-gammalinolenic acid, ACE-I: angiotensin converting enzyme inhibitor, ARB: angiotensin II receptor blocker, ACS: acute coronary syndrome.

**Table 2:** Association between the EPA/AA ratio, EPA, and AA and ACS (multivariate logistic analyses)

	*Total (n = 1733 )*				
	*Smoking status*	*ACS events*	*AOR (per 1 unit increase)*	*95% CI*	*p*
EPA/AA ratio	Never (n = 952)	59	0.20	0.04–0.86	0.03*
	Former (n = 441)	55	1.50	0.44–5.03	0.50
	Current (n = 340)	37	3.73	0.34–40.6	0.27
EPA	Never (n = 952)	59	0.99	0.98–1.00	0.05
	Former (n = 441)	55	0.99	0.99–1.00	0.90
	Current (n = 340)	37	1.01	0.99–1.03	0.14
AA	Never (n = 952)	59	1.00	0.99–1.01	0.41
	Former (n = 441)	55	1.00	0.99–1.01	0.12
	Current (n = 340)	37	1.00	0.98–1.02	0.71
	***Males (n = 1348)***				
	***Smoking status***	***ACS events***	***AOR (per 1 unit increase)***	***95% CI***	***p***
EPA/AA ratio	Never (n = 626)	40	0.22	0.04–1.19	0.07
	Former (n = 418)	50	1.60	0.46–5.46	0.45
	Current (n = 304)	32	0.69	0.03–16.0	0.82
EPA	Never (n = 626)	40	0.99	0.97–1.00	0.08
	Former (n = 418)	50	1.00	0.99–1.01	0.98
	Current (n = 304)	32	1.00	0.98–1.03	0.56
AA	Never (n = 626)	40	1.00	0.99–1.01	0.78
	Former (n = 418)	50	1.01	0.99–1.02	0.07
	Current (n = 304)	32	1.01	0.98–1.03	0.35
			**Females (n = 385 )**		
	***Smoking status***	***ACS events***	***AOR (per 1 unit increase)***	***95% CI***	***p***
EPA/AA ratio	Never (n = 326)	19	0.18	0.009–3.88	0.28
	Former (n = 23)	5	–	–	–**
	Current (n = 36)	5	–	–	–**
EPA	Never (n = 326)	19	0.99	0.97–1.00	0.37
	Former (n = 23)	5	–	–	–**
	Current (n = 36)	5	–	–	–**
AA	Never (n = 326)	19	1.00	0.98–1.02	0.59
	Former (n = 23)	5	–	–	–**
	Current (n = 36)	5	–	–	–**

EPA: eicosapentaenoic acid, AA: arachidonic acid, ACS: acute coronary syndrome, AOR: adjusted odds ratio, CI: confidence interval.

* Statistical significance.

** This model did not converge.

## DISCUSSION

The present results showed a significant inverse association between the EPA/AA ratio and ACS in patients without a smoking history, but not in those with a smoking history.

Smoking is known to adversely affect serum lipid levels. Generally, serum high-density lipoprotein cholesterol is lower, while both low-density lipoprotein cholesterol and TG are higher in smokers than in non-smokers^10^. The serum lipid pattern is a well-known cause of atherosclerosis. In addition, smoking affects the coagulation factors and platelet functions. Fibrinogen is reported to be high in smokers, and this plays an important role in blood coagulation, potentially leading to the development of coronary atherosclerosis. Smoking increases platelet adhesiveness and aggregation, which promote arteriosclerosis and the formation of thrombi, leading to increased ACS prevalence. In addition, as an acute effect, smoking stimulates the sympathetic nervous system via nicotine. This stimulation enhances norepinephrine from the terminal sympathetic nerves and epinephrine from the adrenal medulla that increases the blood pressure and the heart rate^11^.

The EPA/AA ratio is well known as an effective marker for predicting an ACS event, and EPA administration has been proven to have ACS-preventive effects in large-scale clinical studies^12^. EPA is known to exert several preventive effects on arteriosclerosis and ACS, such as reduced TG, increased antiplatelet effects, improved endothelial functions, and reduced blood pressure^6^. However, the present results did not indicate a significant association between the EPA/AA ratio and ACS prevalence in patients with a smoking history. It was not possible to clarify the underlying mechanism for this in the present study owing to the nature of the study; further studies are needed to clarify this mechanism. We hypothesize that smoking may deteriorate the beneficial effects of EPA, such as the antiplatelet effect, antithrombotic effect, and improved endothelial dysfunction and blood pressure, as described above. The balance between EPA and AA, namely the EPA/AA ratio, reflects the manifestation of disease more accurately than the EPA value or AA concentration, because EPA and AA compete with each other when they are metabolized. Therefore, we surmise that smoking may affect the competition between EPA and AA. We suspect that this effect eliminated any possible significant association between the EPA/AA ratio and ACS in patients with a history of smoking.

In the subgroup analyses of the Japan EPA Lipid Intervention Study (JELIS)^12^ that assessed the inhibitory effects of high purity EPA administration against coronary events in Japanese patients with hyperlipidemia, no significant difference was observed in the inhibitory effects exerted by EPA against coronary events between smokers and non-smokers (interaction p=0.89). The JELIS was conducted between 1996 and 1999. The proportions of male subjects in the JELIS and the present study were 31.4% and 77.8%, respectively. In addition, the prevalence of the concomitant use of medications, such as RAS inhibitors and statins, differed between the studies.

The baseline EPA/AA ratio in Japanese populations is relatively higher than that in Western populations owing to the difference in fish consumption patterns^13^. A previous study^14^ conducted on Western subjects reported an average EPA/AA ratio of 0.1, while a mean value of 0.5 was determined for the EPA/AA ratio in the present study.

The main limitation of the present study was its cross-sectional design. Therefore, we were unable to prove any causal relationships or to unbiasedly estimate the incidence measures of ACS. Although the five participating hospitals were representative of urban Tokyo and the characteristics and lifestyles of the visiting patients were largely comparable to residents in this city, they do not represent entire Japan. Thus, the generalizability of our data might be limited. Smoking and/or the EPA/AA ratio may have been affected by gender. Gender-based analyses showed the same tendencies that were observed in all the patients; however, we could not identify any significant differences, mainly because of the small sample size of patients with ACS events. In addition, we did not assess dietary habits in the present study. Future studies are needed to confirm and clarify our findings. Finally, the main conclusion of this study was inferred from a result with a wide range of 95% CI that may be attributable to the relatively small sample size of the study population.

## CONCLUSIONS

We have found that the EPA/AA ratio and ACS occurrence are significantly associated in patients without a smoking history, but not in patients with a smoking history.
